# Genome Sequence of *Lactococcus lactis* subsp. *cremoris* Mast36, a Strain Isolated from Bovine Mastitis

**DOI:** 10.1128/genomeA.00449-15

**Published:** 2015-05-21

**Authors:** Carme Plumed-Ferrer, Simona Gazzola, Cecilia Fontana, Daniela Bassi, Pier-Sandro Cocconcelli, Atte von Wright

**Affiliations:** a Food Biotechnology, Institute of Public Health and Clinical Nutrition, University of Eastern Finland, Kuopio, Finland; b Istituto di Microbiologia, Università Cattolica del Sacro Cuore, Piacenza, Italy

## Abstract

The genome sequence of *Lactococcus lactis* subsp. *cremoris* Mast36, isolated from bovine mastitis, is reported here. This strain was shown to be able to grow in milk and still possess genes of vegetable origin. The genome also contains a cluster of genes associated with pathogenicity.

## GENOME ANNOUNCEMENT

*Lactococcus lactis* and its subspecies *lactis* and *cremoris* are widely used mesophilic dairy starters for the production of cheeses, fermented milks, and vegetable products (1). They are included in the Qualified Presumption of Safety (QPS) list of the European Food Safety Authority (2). However, the number of reports linking *L. lactis* to human and animal infections and clinical disease is increasing (3–8). It is unclear whether *L. lactis*-associated infections are due to the emerging pathogenicity of certain strains of this species or whether the observations are simply a consequence of improved identification. It is also unknown whether the pathogenicity of certain strains is opportunistic due to their ability to simply survive in a host or whether actual virulence factors are involved.

*L. lactis* subsp. *cremoris* Mast36 has already been phenotypically characterized (8) and shows clear differences from dairy starter strains, such as greater ability to ferment carbohydrates and tolerate various stress conditions typically found in the intestinal tract of mammals. Interestingly, these phenotypic properties are also desirable while screening for robust starters that could be used as human or animal probiotics. Thus, a further comparison of *L. lactis* strains is crucial in order to ensure the safety of their use in food industry.

In this work, a *de novo* shotgun sequencing of *L. lactis* subsp. *cremoris* strain Mast36, isolated as the sole bacterium from the milk of a cow suffering from mastitis, was performed. The genome was sequenced using an Illumina HiSeq 1000 platform from the Functional Genomics Centre, Scientific Technological Department of the University of Verona, Verona, Italy. Quality-filtered reads were assembled using the SPAdes software (version 3.1.0) (9), and contig sequences were annotated in the RAST server (10). A 2,610,825-bp assembly was obtained, consisting of a total of 104 contigs and a mean G+C content of 35%. The annotated contigs contain 2,638 putative coding sequences (CDSs) and 60 predicted RNAs. Loaded in the RAST server, the reported genome contains 338 subsystems, which constitute the basis for creation of the *L. lactis* subsp. *cremoris* network.

Comparative analysis between the *L. lactis* subsp. *cremoris* Mast36 genome and other *L. lactis* genomes has revealed a remarkably high similarity with *L. lactis* subsp. *cremoris* KW2, the only sequenced genome of the *cremoris* lineage of plant origin (environmental strain [11]), suggesting that Mast36 is also an environmental strain. However, Mast36 has a cluster of genes coding for proteins that are involved in the metabolism of lactose and galactose uptake and utilization, suggesting an adaptation to grow in milk. Moreover, Mast36 has another gene cluster, which *L. lactis* strains KW2, SK11, and MG1363 (all *L. lactis* subsp. *cremoris*) do not possess, consisting of capsular polysaccharide genes homologous to the *cps* gene cluster in *Streptococcus agalactiae*, the expression of which has been recognized as a major virulence factor (12). Further analyses are in progress to better understand the potential virulence factors unique in this strain.

### Nucleotide sequence accession numbers. 

The whole-genome shotgun project has been deposited at DDBJ/EMBL/GenBank under the accession no. JZUI00000000. The version described in this paper is version JZUI01000000.
